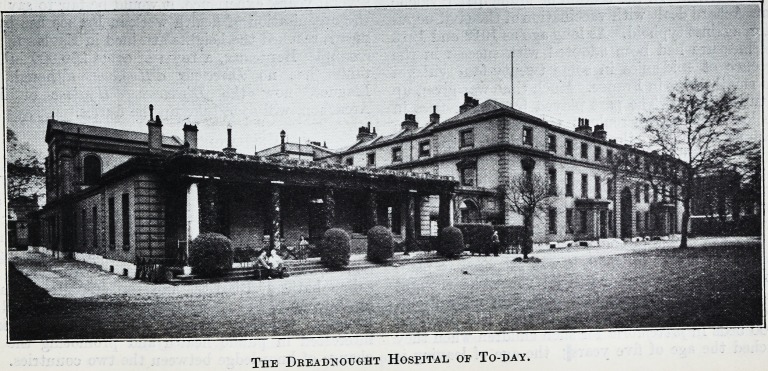# Extending the Seamen's Hospital

**Published:** 1924-07

**Authors:** 


### EXTENDING THE SEAMEN'S HOSPITAL.

### CURING THE SICK SAILOR.

The new Singhanee Ward for Indian Seamen at the Tilbury Hospital will be opened on June 27th by H.R.H. the Duke of York, President of the Seamen's Hospital Society, who will at the same time lay the foundation-stone of the new extensions to the Hospital.

Founded in the winter of 1817 – 18, to administer a fund raised for the relief of the many distressed seamen who were to be found in the streets of London and other large ports, the work of the Society in years gone by was carried on aboard an old battleship, the *Dreadnought*, moored in the Thames off Greenwich, a vessel which had fulfilled her mission as a fighting unit during the Napoleonic Wars and was adapted for the purpose of treating and nursing the men of the mercantile marine. In 1870 the Society removed to the present building on shore —the old Infirmary of the Greenwich Hospital —within whose walls nearly 10,000 sailors received care and treatment in the year 1923. Seafaring men of every nation look to the old *Dreadnought* at Greenwich in their hour of sickness and accident, for they know they will have no difficulty in gaining admission. No "letter" is required, and so they travel thousands of miles, assured that in the care of the Society they will find succour at the hands of devoted doctors and nurses. The Seamen's Hospital Society has many branches. There is a hospital midway between the Victoria and Albert Docks. At Endsleigh Gardens, Euston, is a hospital for the treatment of tropical diseases, to which is attached the London School of Tropical Medicine. At King George's Sanatorium for Sailors, at Bramshott in Hampshire, which owed its inception to Lord Inchcape, relief is sought by sailors who are suffering from consumption, and when treatment at one or other of these institutions is completed patients are often sent to the Angas Convalescent Home in Kent, in order fully to regain their strength before returning to their arduous duties.

[*Elliott & Fry.*

CAPTAIN SIR ARTHUR CLARKE, K.B.E., ELDER BROTHER OF THE TRINITY HOUSE, CHAIRMAN.

At Tilbury Docks there has been erected and equipped a ward devoted solely to the treatment of Lascar seamen. This was made possible by the splendid gift of £6,732 from Mr. Singhanee, a well-known benefactor of Poona, who by this generous act has shown his appreciation of what these brave men do for the overseas trade. This pavilion is attached to the small cottage hospital at Tilbury, which the Society has just taken over. But the demands made upon it by this rapidly developing station of the port are so great that immediate expansion has become imperative. When the new buildings are completed

THE TILBURY HOSPITAL.

there will be beds for forty patients (many of them in private rooms). Only last year the operating theatre was equipped with the latest type of table and other apparatus, and in March of this year the new X-ray department was completed. The cost of the new wards and accommodation for an adequate resident medical and nursing staff will be £40,000, of which only the half has as yet been received. The presence of a hospital thus equipped with all the latest features of medical science will be of the greatest service not only to the seamen who come to the Tilbury Docks, but also to the whole neighbourhood, for this establishment will continue to carry out the work of the former Passmore Edwards District Cottage Hospital, which is now incorporated in it. The Committee of the old hospital have loyally continued to serve on the Committees of the new régime which has been enthusiastically welcomed by the whole locality.

THE OLD DREADNOUGHT.

### JACK ASHORE.

It is a statistical fact that of all sections of the community those who work in ships and shipyards are most liable to accident. It is a truth of human nature that of all men the sailor is the most improvident. Aboard ship he is an expert, but when he comes ashore and has the misfortune to fall ill he often reaches a state of distress not far removed from despair. The State makes no provision for these its servants, and it is only by the generosity of those who realise how much this country owes to the sailor that the work of restoring them to health is carried on.

### THE WORK OF THE CHAIRMAN.

The Chairman of the Society, Captain Sir Arthur Clarke, K.B.E., Elder Brother of the Trinity House and Chairman of the River Committee of the Port of London Authority, has for twenty years used his experience and influence unceasingly in an endeavour to help suffering merchant seamen. This year of Imperial festival at the British Empire Exhibition is a time at which is shown more clearly than ever the importance of the work of the merchant seamen in maintaining the commercial prosperity of the Empire, and no more appropriate time could have been chosen for the opening of the new ward. In that work Sir Arthur Clarke has taken an exceedingly valuable part, both afloat and ashore. A genial Irishman from Cork, in 1891 he was given a command in the Pacific Steam Navigation and Orient lines, and by 1898 had attained the distinction of being an Elder Brother of Trinity House. Sir Arthur is very active on Council Committees of all kinds, and is a member of the Board of Harwich Conservancy, a member of the London Council of the Chamber of Commerce and of the Port of London Authority, and Chairman of the River Committee. He is also Chairman of the Marine Society, Deputy Chairman of King George's Fund for Sailors, and Chairman of the Tropical School of Medicine.

THE DREADNOUGHT HOSPITAL OF TO-DAY.

## Figures and Tables

**Figure f1:**
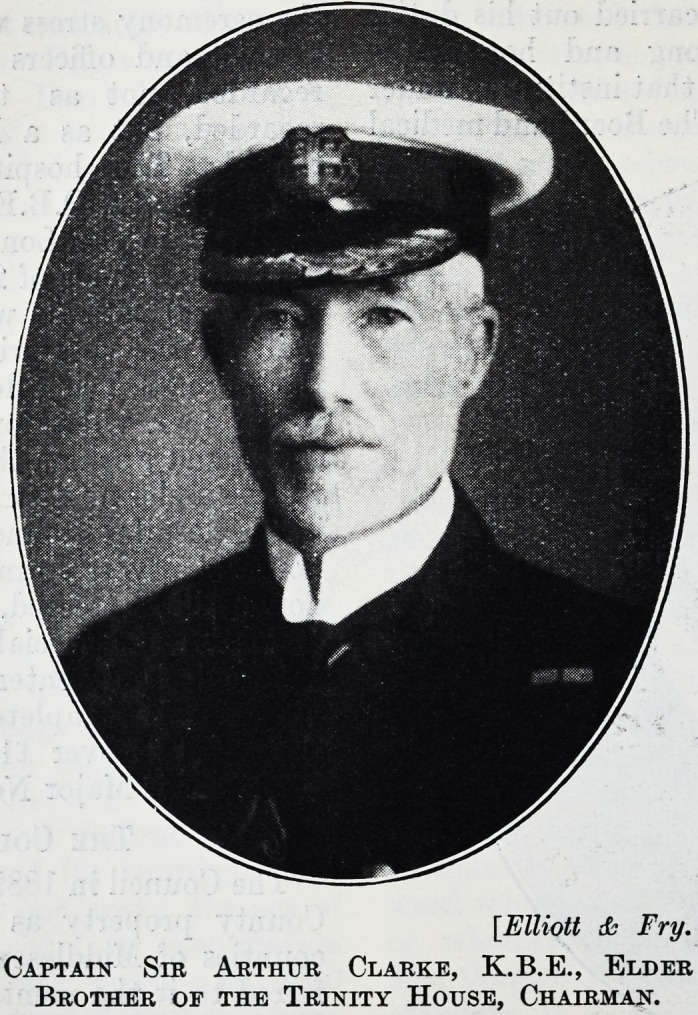


**Figure f2:**
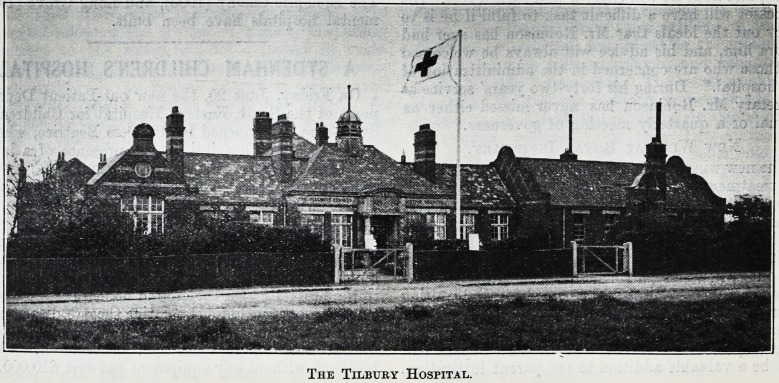


**Figure f3:**
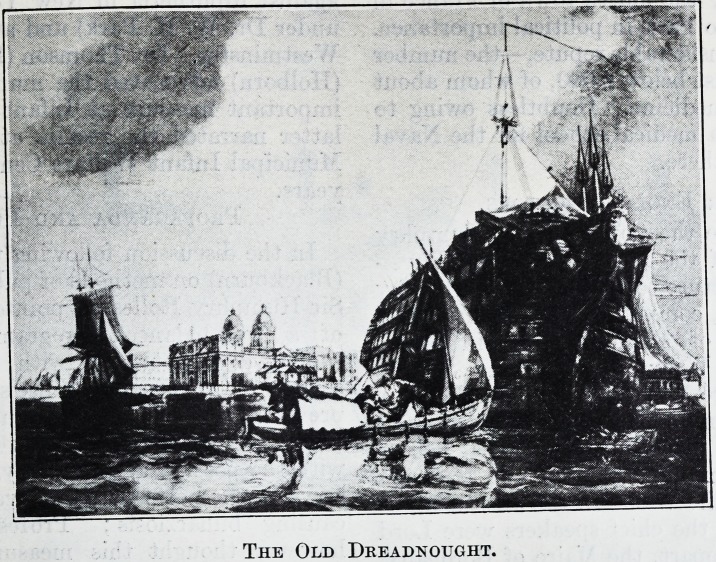


**Figure f4:**